# Molariform Mesiodens in Primary Dentition

**DOI:** 10.1155/2013/750107

**Published:** 2013-03-31

**Authors:** Sachin B. Mangalekar, Tajammul Ahmed, M. Zakirulla, Halawar Sangmesh Shivappa, F. B. Bheemappa, Chandrashekar Yavagal

**Affiliations:** ^1^Department of Periodontology, Maitri Dental College, Chattisgarh, India; ^2^Department of Prosthodontics, Al Jabal Al Gharbi University, Gharyan, Libya; ^3^Department of Pediatric Dentistry, College of Dentistry, King Khalid University, Abha, Saudi Arabia; ^4^Vasant Dada Dental College, Kavalapur, Sangli, Maharashtra, India; ^5^P.M.N.M. Dental College and Hospital, Bagalkot, Karnataka, India; ^6^Department of Pediatric Dentistry, Dr. Hedgewar Dental College, Hingoli, Maharashtra, India

## Abstract

Mesiodens is a midline supernumerary tooth commonly seen in the maxillary arch, and incidence of molariform mesiodens in the maxillary midline is rare in permanent dentition and extremely uncommon in primary dentition. A midline supernumerary tooth in the primary dentition can cause ectopic or delayed eruption of permanent central incisors which will further alter occlusion and may compromise esthetics and formation of dentigerous cysts. This paper reports a rare case of the presence of a molariform mesiodens in the primary dentition. On clinical and radiographic examination, flaring of the primary central incisors was seen, with a molariform mesiodens consisting of multiple lobes or tubercles on the occlusal surface with the well-formed root. The treatment plan consisted of the extraction of the supernumerary tooth and regular observation of permanent central incisors for proper eruption and alignment.

## 1. Introduction

The term mesiodens refers to a supernumerary tooth present in the midline of the maxilla between the two maxillary central incisors. The occurrence of mesiodens in primary dentition is quite rare despite the fact that in permanent dentition it has even been considered as the most common dental abnormality [1]. The reported prevalence in general population ranges between 0.15% and 3.8% in the permanent dentition whereas in the primary dentition, it ranges between 0% and 1.9% [2, 3]. No significant sex distribution is noted in the primary supernumerary teeth [4]. It has been believed that environmental factors along with hereditary factors are combined to cause the condition [5]. However, the presence of multilobed or tuberculate form of mesiodens in the deciduous dentition is extremely rare and is mentioned only once or twice in the literature [6]. 

## 2. A Case Report

**Figure 1:**
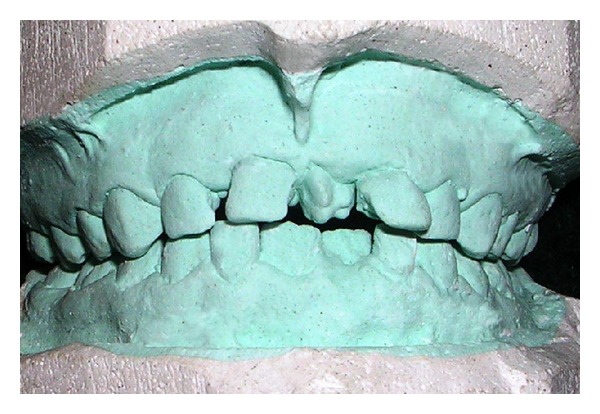
Model showing multilobulated mesiodens between maxillary primary central incisors.

**Figure 2:**
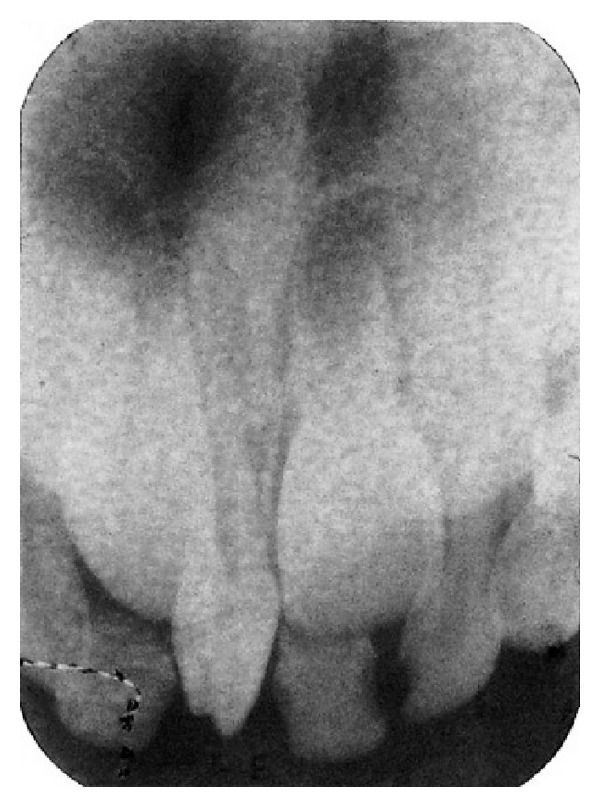
IOPA radiograph showing multilobulated mesiodens between maxillary primary central incisors.

A six-year-old girl along with her mother reported to the department with the complaint of a tooth with an unusual appearance in the upper front teeth area. On intraoral examination, a mesiodens was noticed in the maxillary arch with multiple lobes or tubercles on the occlusal surface. Patient had full complement of deciduous teeth with no other anomalies. Medical and family history was not relevant and noncontributory. Luten's criteria [7] and Howard's classification [8] were used to carefully evaluate the supernumerary tooth which revealed four separate lobes with well-formed developmental grooves on the occlusal surface of the mesiodens which could be clearly distinguished. The presence of the tuberculate mesiodens resulted in reduced space in arch between the central incisors causing flaring of them (Figure 1). On examination of the central incisors, they exhibited grade-II mobility and neared exfoliation. There was no interference of the mesiodens in occlusion. Radiographic examination with periapical radiograph revealed completely formed root and the presence of multiple cusps that were well demarcated on the radiograph with resorption of incisors roots (Figure 2). 

**Figure 3:**
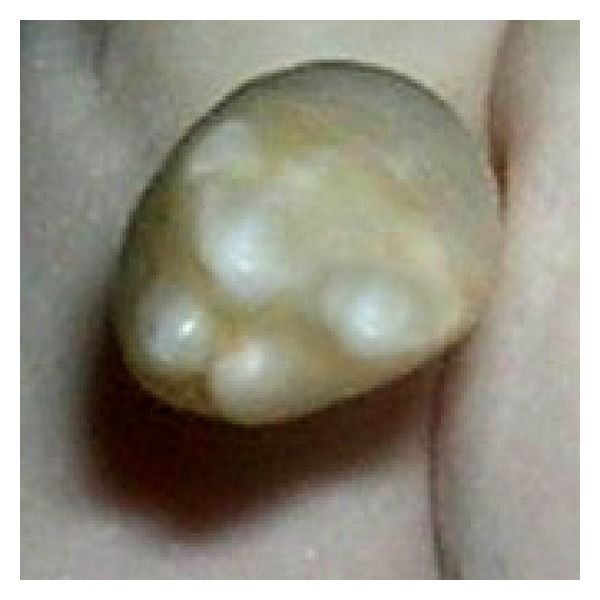
Molariform mesiodens after extraction.

Based on the clinical and radiographic examinations, the supernumerary tooth was diagnosed as a multilobed tuberculate mesiodens in the deciduous dentition. A comprehensive treatment plan was formulated, which included extraction of the deciduous central incisors and the tuberculate mesiodens under local anaesthesia (Figure 3). Their presence would have significant impact on the eruption and position of the permanent central incisors and possibility of future malocclusion.

## 3. Discussion

The presence of the mesiodens in the permanent dentition is well documented. Mesiodens in the permanent dentition show great variation and are classified accordingly. 

Possible explanation for the less frequent reporting of deciduous supernumerary teeth includes infrequent detection by parents, as the spacing frequently encountered in the deciduous dentition may be utilized to allow the supernumerary tooth or teeth to erupt with reasonable alignment. Also, many children have an initial dental examination following the eruption of the permanent anterior teeth so anterior deciduous teeth which have erupted and exfoliated normally would not be detected [9].

Numerous classifications have been proposed in the literature to classify supernumerary with varied acceptance. No single classification is adequate and is used according to the convenience.

The classifications are based on location; morphology, axial inclination, and other criterion are used for classification of such teeth. Sometimes the presence of supernumerary teeth is seen in developmental syndromic cases such as cleft lip, and palate, cleidocranial dysostosis, Down's syndrome and Gardener's syndrome. Other cases where syndromic involvement is seen are enumerated in Box 1.

The aetiology of the supernumerary teeth is not clearly understood despite its regular presentation. Various theories have been postulated to explain the same. Atavistic theory states that mesiodens represented a phylogenetic relic of extinct ancestors who exhibited three central incisors [10]. Another theory suggests that the supernumerary tooth is a result of dichotomy of the tooth bud; others suggest that they are the result of local independent conditioned hyperactivity of dental lamina. Heredity can also play a pivotal role in the formation of the supernumerary teeth sometimes associated with or without syndromes. Association of supernumerary teeth is also seen with cysts like dentigerous cysts and odontomes [11]. The presence of multilobed mesiodens with palatal talon cusp is also reported in the literature [12].

Management of patients with supernumerary teeth may vary from simple extractions coupled with orthodontic therapy to attain a good occlusion as well as aesthetics. Occasionally, retaining such teeth would be prudent where space considerations impacted supernumerary without any problems; in such cases simple routine followup is required. 

Multiple lobulated mesiodens with developmental grooves can present with a problem in terms of maintaining a proper oral hygiene which would invariably lead to development of caries. Complications that are associated with mesiodens are enumerated in Box 2.

In the present case, the erupted multilobulated mesiodens in the 6-year-old girl was a great aesthetic concern to the patient and their family members; however, the presence of the multilobulated mesiodens in the primary dentition is extremely rare. Only one case in the literature was reported by Sharma [13]. In the present case, the crown of the mesiodens presented with unusual crown morphology with 4 separately well-formed lobes with developmental grooves and full formed root. Failure to identify or not treating would have resulted in a deep impact on the child causing psychological trauma and imitate malocclusion.

## 4. Conclusion

A careful examination by clinical and radiographic methods can reveal a rare manifestation of the presence of hyperdontia, and they can cause and increase the incidence of malocclusion. No single tailored treatment is available. Treatment planning can be done based on the clinical case. In the present case, the presence of such teeth would have created a different path of eruption of the permanent central incisors and would have resulted in deranging the occlusion and poor aesthetics for the child.

## Figures and Tables

**Box 1:**
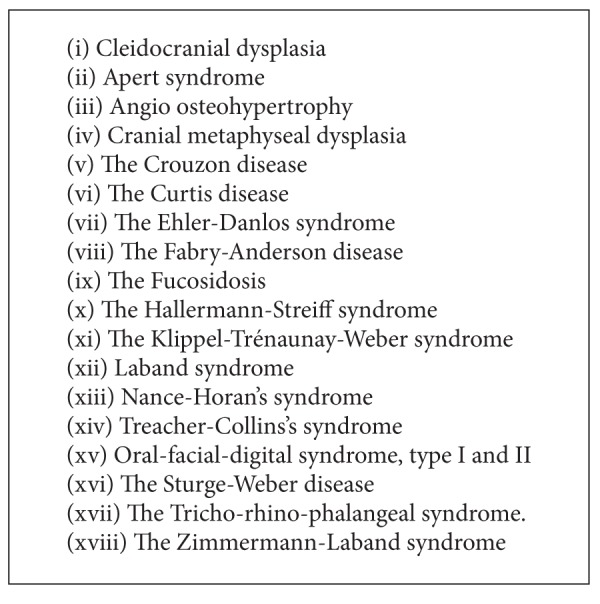
Supernumerary teeth associated with syndromes.

**Box 2:**
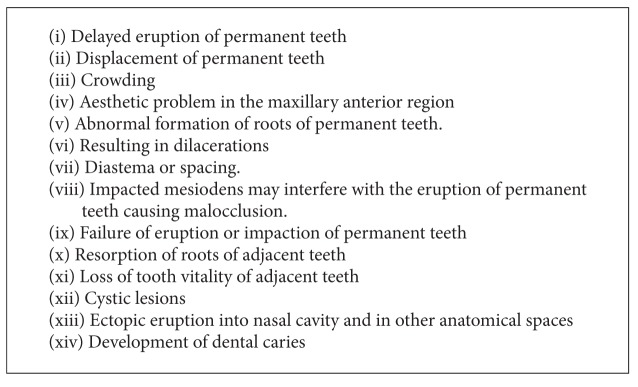
Complications that are associated with mesiodens.
